# Recent Developments in Chitosan-Based Adsorbents for the Removal of Pollutants from Aqueous Environments

**DOI:** 10.3390/molecules26030594

**Published:** 2021-01-23

**Authors:** Daniele C. da Silva Alves, Bronach Healy, Luiz A. de Almeida Pinto, Tito R. Sant’Anna Cadaval, Carmel B. Breslin

**Affiliations:** 1 Department of Chemistry, Maynooth University, W23 F2H6 Maynooth, Co. Kildare, Ireland; DANIELE.COSTADASILVAALVES.2021@MUMAIL.IE (D.C.d.S.A.); bronach.healy.2017@mumail.ie (B.H.); 2 School of Chemistry and Food, Federal University of Rio Grande, Rio Grande, RS 96203-900, Brazil; dqmpinto@furg.br (L.A.d.A.P.); titoeq@gmail.com (T.R.S.C.J.)

**Keywords:** chitosan, adsorbent, carbon, graphene oxide, silica, magnetic separation, dyes, heavy metals, adsorption, Langmuir isotherm

## Abstract

The quality of water is continuously under threat as increasing concentrations of pollutants escape into the aquatic environment. However, these issues can be alleviated by adsorbing pollutants onto adsorbents. Chitosan and its composites are attracting considerable interest as environmentally acceptable adsorbents and have the potential to remove many of these contaminants. In this review the development of chitosan-based adsorbents is described and discussed. Following a short introduction to the extraction of chitin from seafood wastes, followed by its conversion to chitosan, the properties of chitosan are described. Then, the emerging chitosan/carbon-based materials, including magnetic chitosan and chitosan combined with graphene oxide, carbon nanotubes, biochar, and activated carbon and also chitosan-silica composites are introduced. The applications of these materials in the removal of various heavy metal ions, including Cr(VI), Pb(II), Cd(II), Cu(II), and different cationic and anionic dyes, phenol and other organic molecules, such as antibiotics, are reviewed, compared and discussed. Adsorption isotherms and adsorption kinetics are then highlighted and followed by details on the mechanisms of adsorption and the role of the chitosan and the carbon or silica supports. Based on the reviewed papers, it is clear, that while some challenges remain, chitosan-based materials are emerging as promising adsorbents.

## 1. Introduction

Improving water quality is one of the major environmental challenges worldwide to be solved, since water resources are increasingly scarce due to population growth, climate change and increased demand for water in industrial and agricultural activities [1]. In addition, the inappropriate disposal of organic and inorganic contaminants combined with disinformation and neglect in the treatment of these compounds can result in irreversible damage to the aquatic environment and, consequently, to humans [2,3]. Dyes, phenolic compounds, metallic ions and micropollutants, such as pesticides and drugs, have all been detected in wastewaters, surface and even drinking water, indicating that the conventional methods used in treatment plants are not optimised for their removal [4]. Consequently, the removal of these pollutants with high toxicity, even when present at low concentrations, has been increasingly studied in the scientific world [5,6].

Several techniques have been developed based on hybrid systems [7], membrane filtration [8] and biological degradation [9] to reduce the content of pollutants in water. However, the slow response, sensitivity and high energy demand are some of the disadvantages of such techniques. In addition, they are not very efficient when the effluent has a low content of suspended colloidal particle and a high load of organic matter. A promising alternative to the treatments mentioned is adsorption, due to its simplicity of operation and effectiveness [10]. Therefore, the search for new adsorbent materials that can be used to remedy aquatic contamination has been stimulated. The biopolymer chitosan is attracting considerable interest as a matrix for adsorbent material development, since this biopolymer has a high density of hydroxyl groups (–OH) and primary amines (–NH_2_) that act as active adsorption sites, making it an efficient adsorbent [11].

Chitosan (β–(1–4)–d–glucosamine) is a polysaccharide that possesses two types of monomers, one containing an acetamido group (2–acetamido–2–deoxy–β–d–glucopyranose residues), and another containing an amino group (2–amino–2–deoxy–β–d–glucopyranose residues). Chitosan is not available directly in the environment, it is obtained from chitin (β–(1–4)–*N*–acetyl–d–glucosamine), usually by the alkaline or enzymatic *N*-deacetylation of chitin [12,13,14]. The characteristic that differentiates the structure of chitin and chitosan is the substitution of the acetamide group at position 2. This directly influences the solubility properties of these compounds, with chitin being insoluble and inert, and chitosan soluble in weak acids [13,15].

Currently, chitin and chitosan are produced commercially in Japan, United States, India, Poland, Australia, and Norway, and to a lesser extent, in Canada, Italy, Chile and Brazil [16,17]. The annual production of chitin in nature has been estimated to be as high as 1 × 10^10^ to 1 × 10^12^ tonnes [18], which makes this biopolymer a cheap and available resource. However, as the biodegradation rate of chitin is slow, the production of high amounts through shrimp and seafood processing creates an environmental problem, and the conversion of this waste material into high-value products, such as chitosan, can be an attractive solution [19].

During the past few decades, many researchers have focused on the development of chitosan-based materials to solve problems in environmental and biomedical engineering fields, and in the development of innovative products for other different applications [20,21,22]. From 2010 to 2015, more than 15,000 articles and about 20 books on chitosan were published worldwide. This ever increasing interest is connected with the attractive properties of chitosan, such as biodegradability, low toxicity and biocompatibility, coupled with the availability of natural resources required for its chemical and enzymatic modifications for specific end uses [19,23]. Chitosan has been used in many fields such as food, medicine, cosmetics and wastewater treatment [24]. In addition, the efficient utilisation of marine biomass resources has become an environmental priority that leads to intensified research on chitosan production and its applications [25].

In this review, the applications of chitosan-based materials as adsorbents for the removal of pollutants from aqueous environments are reviewed. Although there are a number of review articles that describe the environmental applications of chitosan [19,24,26,27], in this review we focus initially on the sources, properties and chemical modifications of chitosan and discuss the various factors that influence its properties as a material for the removal of pollutants. Next, we review the support materials utilised and combined with chitosan, including the emerging carbon and silicon supports, providing a more comprehensive account than previously published.

## 2. Chitosan

Chitin is the second most abundant natural polysaccharide (biopolymer) on Earth, following cellulose [26]. In 1811, the first chitin was extracted by Henri Bracon from mushrooms and was named initially as “fungine”. In 1823, Odier [28] found the same material in the insect exoskeleton and called it chitin. Later, chitin was found in crab shells, confirming that it can be found in the crustacean exoskeleton. In 1859, Charles Roughet discovered that chitin could be transformed into a water-soluble form after chemical modification [19]. In 1894, this chemically modified chitin was called chitosan by Hoppe-Seyler [29,30]. Although chitin was discovered 30 years before cellulose, most of the research was focused on cellulose, due to the high investments made by the textile industries. Thus, chitin and chitosan remained restricted only to basic research during this period. Around 1970, interest in natural products increased and investigations directed at exploring the potential applications of chitosan began to attract considerable attention [13]. In 1977, the “1st International Conference on Chitin and Chitosan” was organised in Boston, USA, where the scientific and industrial communities leveraged the world’s interest in these biopolymers [23].

### 2.1. Source and Production

**Figure 1:**
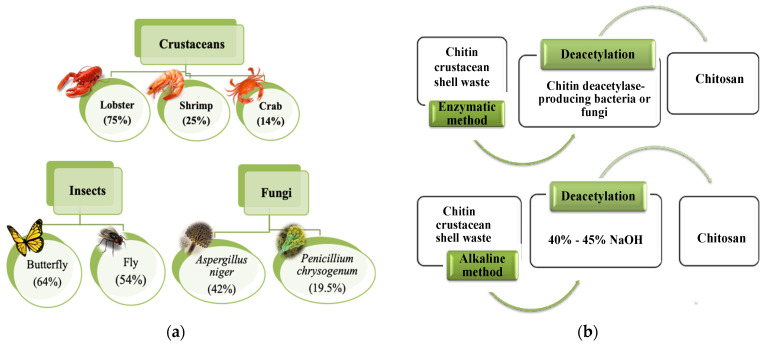
(**a**) Examples of chitin content from different sources, (**b**) Chitin deacetylation methods to produce chitosan.

Chitosan is derived from chitin, usually obtained from natural sources such as the residues of shrimp, crab and lobster, fungal mycelia and green algae [15,31,32]. This biopolymer can also be found in the exoskeletons of insects and fungal cell walls, as shown in Figure 1a. Chitin content in fungi varies between 19% and 42%, compared to the exoskeleton, where chitin can reach up to 75% [29,33]. Not all shellfish wastes are good sources of chitin. The blue crab (*Callinectes*) contains 14% of chitin, while oyster and clam shells have chitin content in the range of 4–6% [29,34]. Chitin content can vary with the proportion of minerals, proteins, and carotenoids, depending on the species, reproductive cycle phase, nutritional status, age, and also the peeling conditions during the processing [13,17]. Chitosan can be obtained by deacetylation of chitin through enzymatic or alkaline methods (Figure 1b). However, the enzymatic method has been limited only to the laboratory scale, while the alkaline method has been more widely used on an industrial scale, due to its short processing time, simplicity, and low operational costs [29].

#### 2.1.1. Enzymatic Methods

The first step in the production of chitosan is the extraction of chitin from the seafood waste and this can be achieved using both biological and chemical approaches. The biological process involves the use of bacteria, that produce organic acids, and enzymes for the demineralisation and deproteinisation of crustacean shells [35]. In the demineralisation step, the lactic acid produced by bacteria reacts with the calcium carbonate component in the crustacean biomass waste resulting in the formation of calcium lactate, which can be precipitated and removed by washing, while proteases from the bacteria eliminate proteins [35]. *Bacillus cereus* A21 has shown high activity with both the demineralisation and deproteinisation steps from shrimp shell wastes, 91% and 80%, respectively [36]. Likewise, the deproteinisation and demineralisation from natural crab shell wastes by *Bacillus pumilus*A1 were 94% and 80%, respectively [37]. These results suggest that the chitin production process by the enzymatic method of the seafood wastes could be applicable and become a friendly environmental alternative.

The deacetylation step that gives rise to the production of chitosan can be promoted by chitin deacetylase. This enzyme was first found in *Mucorrouxii* (*Zygomycetes*) in 1974, by Araki and Ito [38]. In 1984, Davis and Bartnicki-Garcia found evidence that changes in the culture medium during the fungi growth phase can directly influence the production of chitosan [39]. The reaction mechanism of the chitin deacetylase from *Mucorrouxii* is considered as a multipoint attack mode; specifically, the enzyme systematically hydrolyses acetyls from the non-reducing end of the binding site after it binds to a substrate chain, and then leaves the substrate and binds to another one [40]. Strains that produce a large amount of extracellular deacetylase with high activity are very valuable in the production of chitin deacetylase and the production of chitosan. Nevertheless, there are still some problems such as low enzyme activity and low yields of deacetylase-producing strains. Moreover, natural chitins are crystals, not a good substrate for deacetylase. Hence, many preparations still need to be carried out before the chitin deacetylase method can be used in the industrial production of chitosan [41].

Chitin deacetylase-producing bacteria, such as *Serritia* sp. and *Bacillus*sp, may replace the current fungal strains. This microorganism culture method is another possibility to obtain chitosan, removing the acetyl groups by catalysing the substrate with the enzymes produced by these microorganisms. Moreover, bacteria grow faster than fungi in large-scale fermentation processes. Recently, research has concentrated on the breeding of microorganism strains and optimisation of the culture medium. Chitosan formed by this method has shown good ion adsorption capacity [41], making it suitable for environmental applications.

#### 2.1.2. Alkaline Methods

The traditional chemical methods to extract chitin from crustacean shell wastes involve three steps: demineralisation, deproteinisation and decolourisation. In the first step, crustacean shells are washed, dried, and grounded to smaller sizes [42]. In order to remove mineral constituents, mainly calcium carbonate, the powdered raw material is treated with dilute hydrochloric acid followed by the precipitation of calcium chloride. Alkali treatment is used for deproteinisation of the demineralised shells. Proteins are eliminated through solubilising with dilute aqueous sodium hydroxide and in the process, N-acetyl groups within the polymer backbone are hydrolysed. The recovery of protein may be obtained by lowering the pH to about 4.0. An additional decolourisation step can be incorporated when a colourless product is required. Acetone or organic solvent mixtures are used to remove the pigments such as carotenoids [15].

**Figure 2:**
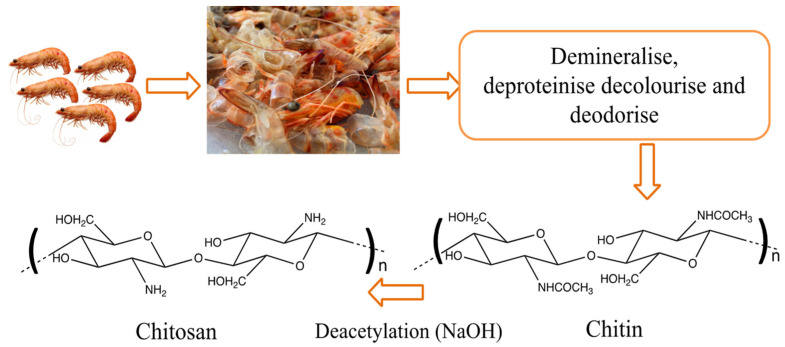
Process of obtaining chitosan by the deacetylation alkaline treatment of chitin from shrimp shell wastes.

Chitosan is obtained by deacetylation of chitin in 40–45% sodium hydroxide, as shown in the sketch of Figure 2. The alkaline treatments hydrolyse the acetyl groups and transform the *N*–acetyl–d–glucosamine units into d–glucosamine units with free NH_2_ groups [15]. Chitosan, with different degrees of deacetylation, is generated depending on the reaction temperature, time, and concentration of the alkaline solution [43]. An additional purification step can be performed to obtain high purity chitosan. For this, the deacetylated product is dissolved in acid, centrifuged or filtered, and the chitosan is precipitated through the addition of alkali generating pure chitosan (90–95%) in paste form. These steps can be repeated to obtain higher purity chitosan (purity >99.9%) [15,23].

The drying of chitosan is an important step in its production. In general, after drying, the desired product should contain a moisture content lower than 10% (wet basis), to ensure good physicochemical and microbiological aspects during prolonged storage. Polymerisation and Maillard reactions are the main alterations that should be avoided during the drying operation [44]. Chitosan is composed mainly of carbohydrate monomer units that, at high temperatures, are capable of undergoing caramelisation of the polymer. Therefore, in this process, one of the key parameters is temperature [45,46]. Different techniques have been used to obtain good quality dried chitosan, such as spouted bed drying [47], spray drying [48], convective tray drying [49], oven drying and infrared drying [50], lyophilization [51] and low-pressure superheated steam drying [52]. All these techniques have shown that the most important factors are temperature and residence time, which must be controlled to obtain a high-quality product. For example, Dotto et al. [47] have shown that an increase in the temperature (from 90 to 100 and 110 °C), using a spouted bed drying technique, causes an increase in powder darkening, an increase in molecular weight (from 147 to 25 kDa) and increased particle size (from 100 to 200 μm). Hence, the best powder quality was obtained at 90 °C, which resulted in the final humidity content being within the commercial range (10%).

### 2.2. Structure and Properties

**Figure 3:**
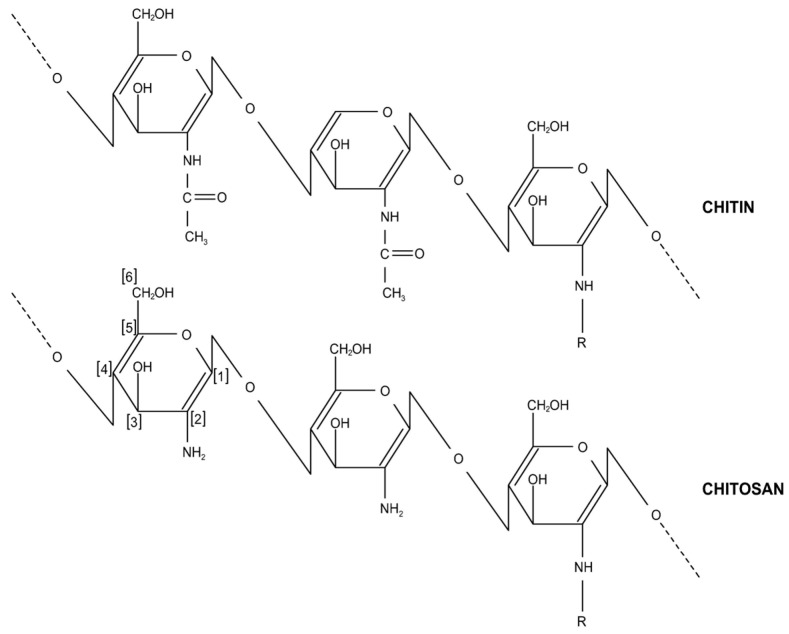
Chemical structure of chitin and chitosan.

Chitosan, a partially deacetylated product of chitin, is a biopolymer composed of β–(1–4)–d–glucosamine, as shown in Figure 3. This biopolymer is a glycosaminoglycan and consists of two common sugars, β–(1–4)–2–acetamido–d–glucose and β–(1–4)–2–amino–d–glucose, glucosamine, and *N*–acetylglucosamin, respectively. The proportion of each depends on the alkaline treatment, and usually deviates from an equal contribution. In terms of structure, chitosan is analogous to cellulose, in which the hydroxyl (in cellulose) has been replaced by acetamido or amino groups (in chitosan) at carbon-2 [53]. Thus, unlike other polysaccharides abundant in carbon, oxygen, and hydrogen; chitin and chitosan contain additional nitrogen (6.89%), making them interesting commercially [54].

The physicochemical properties of chitin and chitosan strongly depend on molecular chain orientation and regular packing. The abundance of hydroxyl groups and highly reactive amino groups in chitosan or its N-acetyl counterpart with a strong tendency for intra- and inter-molecular hydrogen bonding, results in the formation of linear aggregates and rigid crystalline domains. However, chitosan is usually less crystalline than chitin, which presumably makes chitosan more accessible to reagents and, consequently, more soluble. Most of the aqueous acids dissolve chitosan whereas chitin is soluble in very few solvents. The protonation of amino groups by acids along the chitosan chain creates a multitude of cationic sites, which increases its solubility by increasing the polarity. This unique property expands the potential applications of chitosan, including its ability to adsorb different pollutants. Amine groups, for example, are strongly attracted to metal ions due to the lone pair of electrons on the nitrogen atoms [55,56]. The protonation of these amine groups may lead to the electrostatic attraction of anionic compounds, such as anionic dyes [57] and halogens [58]. Moreover, the existence of these free –NH_2_ and −OH active groups allows the adsorption of other pollutants, such as, phenol [59,60], antibiotics [61,62] and pesticides [63]. Hence, the chitosan adsorption capacity depends on its crystallinity, affinity to water, and deacetylation percentage [64].

The main properties of chitosan are summarised in Table 1. Some of the intrinsic properties of chitosan, such as its polycationic character in acid media, its ability to form hydrogen bonds, van der Walls and electrostatic interactions, make it an efficient adsorbent material. Other characteristics, such as the degree of deacetylation (DD), crystallinity, molecular weight (MW), solubility, surface area and particle size will all influence the properties of the final chitosan-based material and its adsorption potential [23]. Therefore, these properties and their optimisation are central in the formation of efficient adsorbent materials.

#### 2.2.1. Deacetylation Degree

The deacetylation degree (DD) of chitosan is one of the most important parameters as it defines the acetyl content in the biopolymer and it can be increased by repeating or prolonging the alkaline treatment step in the chitin deacetylation process [15]. It can be easily determined using several analytical tools, including UV spectrophotometry [65], X-ray diffraction [66], FTIR-spectroscopy [67,68] and titration methods [69,70,71]. Increases in DD lead to an increase in the number of free amino groups on the chitosan polymeric chain. These amino groups are responsible for differences in the physicochemical properties and structure of chitosan, due to intra- and inter-molecular hydrogen bonds. As a consequence, the chitosan solubility and polycationic character are increased, expanding the applications of chitosan [72].

The control and manipulation of the physicochemical properties of chitosan, such as the mechanical properties, crystallinity, swelling and thermal degradation, have been shown to correlate with the distribution of the acetyl groups along the main chain [73,74]. With an increase in the DD, the charge density along the chain increases and the chitosan chain becomes more flexible, tending to form a random coil with more inter- and intra-molecular hydrogen bonds within the chain. In Table 2, the influence of DD on the physicochemical properties is shown, where it is seen that the DD % has a significant impact.

In most cases, an increase in DD was shown to result in an increase in tensile strength and crystallinity and a decrease in the percentage of elongation of the materials. This effect of increasing tensile strength with increasing DD is usually attributed to an increase in crystallinity of the chitosan. Chitosan chains with higher DD have fewer acetyl side groups leading to a more efficient and regular packing of the polymer chains, which in turn, promotes crystallinity in the chitosan [77,85,86]. On the other hand, chitosan with lower DD presents more acetyl side groups that prevent regular packing of the chains due to steric hindrances leading to a reduced crystalline or an amorphous structure [77,86]. Despite the improved tensile strength and stiffness of the chitosan which is observed on increasing the crystallinity this also leads to an increase in the brittleness and a decrease in the percentage of elongation [86]. For example, Zhuang et al. [86] evaluated chitosan films with different DD (81.0%, 88.1% and 95.2%). They reported that the tensile strengths of chitosan films increased from 28.86 to 32.96 MPa and the elongation decreased from 54.31% to 41.66% as DD increased from 81.0% to 95.2%. In applications where film-formation properties of chitosan are important, chitosan with improved tensile strength is an advantage [81,87]. Liu et al. [88] developed composite films of gelatin and chitosan of different MW and DD, and evaluated the interactions between the two polymers in order to improve the films produced. It was verified that the tensile strengths of gelatine films were improved, especially when using chitosan of higher DD and MW. On the other hand, Moura et al. [89] reported a decrease in tensile strength and in elongation of chitosan films with increasing DD, while Nunthanide et al. [75] found both an increase and decrease in tensile strength and elongation, depending on the molecular weight, on increasing DD. These studies which account for the role of MW in the observed results are interesting and highlight the role of both MW and DD.

DD also influences the swelling and thermal degradation characteristics of chitosan [77,78,90,91]. These studies have shown that chitosan with higher DD exhibits faster thermal degradation rates and reduced swelling, as compared to lower DD chitosan. These characteristics may also depend on crystallinity. Chitosan with higher DD and crystallinity are expected to have a close-packed microstructure, which limits water permeation and thus reduced swelling [77]. Moreover, a decrease in the N-acetyl content results in a decrease in the thermal stability as the N-acetyl domains are more thermally stable than the deacetylated ones [92]. Khoulenjani et al. [90], in using chitosan with different DDs (56%, 64% and 74%), showed that the swelling index decreased from 216% to 115% with an increase in DD from 56% to 74%. Nunthanide et al. [75] also reported that the films become more brittle with a lower swelling index with an increase in DD. Wanjun et al. [93] verified that an increase in DD resulted in a decrease in the thermal stability of chitosan due to the decreased acetyl content. This relationship between thermal effects and the DD of chitosan has also been confirmed by Kittur et al. [76]. In another study, Tavares et al. [84] demonstrated that the DD of chitosan had a positive influence on the thermal degradation behaviour. They prepared genipin-crosslinked chitosan beads and evaluated the effect of the chitosan DD (83%, 94% and 96%) on their characteristics. It was verified that the chemical interactions between chitosan and genipin result in a material more thermally stable, especially when a higher chitosan DD (96%) was used. This behaviour was attributed to the decrease in the hydrophilic groups available to form hydrogen bonds with water molecules, resulting in a material more thermally stable.

However, the highly hydrophilic character of chitosan with high DD might be a disadvantage for its surface modification and hence limit the development of chitosan-based materials. Iamsamai et al. [94] have shown that the DD of chitosan plays a critical role in the dispersion of multiwall carbon nanotubes (MWCNTs) and their stability. They confirmed that the chitosan surface coverage on the MWCNTs was twice as high when modifying the surface of the nanotubes with the 61% DD than when using the 93% DD chitosan; suggesting that the dispersion of MWCNTs with chitosan might be improved when using chitosan having a lower DD level.

In addition to the above properties, DD also affects the adsorption properties of chitosan-based materials, since it is linked directly to its cationic properties. Piccin et al. [95] studied the adsorption of FD&C Red 40 dye by chitosan powder with different deacetylation degrees. It was shown that an increase in the DD from 42% to 84% caused an increase in the adsorption capacity from 266 to 373 mg g^−1^. Habiba et al. [96] prepared a chitosan/polyvinyl alcohol/TiO_2_ composite with different DD for methyl orange adsorption. They have shown that the adsorption capacity was higher for the composite containing chitosan with higher DD. Furthermore, the composite containing chitosan with higher DD was more reusable and stable with good adsorption capacity even after 15 regenerations. Józwiaket al. [97] have developed chitosan adsorbents with different forms (flakes and hydrogel granules) and different DD (75%, 85% and 90%) to remove Reactive Black 5 (RB5) from aqueous solutions. The highest adsorption capacity (1559.7 mg g^−1^) was obtained for the chitosan-hydrogel granules formed with 90% DD. Chitosan hydrogel granules reached up to 224% higher adsorption capacity (q_DD75%_ = 1307.5 mg g^−1^) than chitosan in the form of flakes (q_DD75%_ = 403.4 mg g^−1^), which indicated that the chitosan form is also important to the adsorption operation. Besides, the DD of chitosan had a particularly large impact on the RB5 adsorption effectiveness of chitosan in the form of flakes. The adsorption on the flakes with a 90% DD was 1049.6 mg g^−1^ and was higher by 260% than on the flakes with 75% DD. All the authors of these studies concluded that the DD had influenced the physicochemical properties as well as the interactions of chitosan with the pollutants in the adsorption process. Therefore, DD is a major factor in matching chitosan to other materials to develop potential adsorbent materials [98].

#### 2.2.2. Molecular Weight

The molecular weight (MW) of chitosan is a characteristic associated with the number of monomeric units per polymer molecule (n). The deacetylation process brings about a change in MW and depending on the source and preparation procedure, the average MW of chitosan may range from 50 to 2000 kDa [99]. The MW of chitosan can be measured by light scattering, high-performance liquid chromatography (HPLC) and viscosimetric methods [23]. Chitosan can be classified as low molecular weight (LMW), medium molecular weight (MMW) and high molecular weight (HMW) [99]. Generally, the MW of chitosan can be modified by using depolymerisation techniques where the high MW chitosan is converted to a lower MW. These MW modifications are important as they can preserve the integral structure of chitosan [99,100].

The control, evaluation and modifications of this characteristic are fundamental since MW affects many of the physicochemical properties, including solubility, viscosity, crystallinity, tensile strength, adsorption and elasticity. Consequently, MW has a significant effect on the applications of chitosan. Zhou et al. [73] prepared hydrogels with different MW of chitosan and verified that the viscosity of the hydrogels increased with MW, increasing from 88 to 1360 kDa at 37 °C. Moreover, the increase of MW was favourable for sol-to-gel transition and high molecular chitosan was optimal for hydrogel preparation. In Table 3, the various molecular weights employed in chitosan-based materials are summarised. In all cases, the MW is a key factor that influences the tensile strength (TS) and elongation-at-break (EB) properties, as well as the different physical forms of chitosan.

Zhong et al. [101] studied the effect of MW on the properties of chitosan films and found that the conductivity, viscosity, surface tension, and crystallinity of the chitosan film were raised with increasing MW due to an increase in the proportion of amine-groups and degrees of chitosan chain entanglements. Moura et al. [89] verified that the tensile strength, elongation-at-break and water barrier properties of chitosan films were improved with an increase in MW. On the other hand, Ziani et al. [102] showed that the low MW films exhibited greater tensile strengths and percentage of elongation compared to the high MW films despite the high DD of the low MW chitosan. In this study, they verified that the MW had more influence on the mechanical properties than DD. These characteristics were attributed to the number of hetero-monomers, which form stronger films than the character of the acetylated or deacetylated monomers. In general, according to Table 2 and Table 3, several studies have demonstrated that DD and MW can be used to manipulate the physical-mechanical and the thermal degradation properties of chitosan materials. However, these studies also highlight that there is a significant and complex interaction between DD and MW and this interaction can lead to conflicting results, e.g., tensile strengths have been shown to increase and decrease with an increase in DD, according to the MW, and increasing MW can both increase and decrease the percentage of elongation, depending on DD. It is also noted that the type or mode of fabrication of the chitosan (e.g., films, gels, membranes, etc.) may be further influenced by the DD and MW properties.

**Table 3:** Tensile strength (TS) and elongation-at-break (EB) properties of chitosan-materials with different molecular weight (MW) chitosan.

Material	Chitosan MW (kDa)	TS (MPa)	EB (%)	Ref.
Chitosan film	6.55	8.67 ± 1.72	32.53 ± 4.78	[101]
	12.93	12.05 ± 2.24	35.52 ± 6.32	
	47.70	11.51 ± 2.25	25.74 ± 3.69	
Chitosan coated cellulose paper	25.00	9.70 ± 1.50	6.7 ± 1.3	[103]
	2100	13.40 ± 1.50	6.9 ± 0.5	
Chitosan film	101.0	22.30 ± 0.2	8.7 ± 0.2	[89]
	153.6	29.50 ± 0.1	11.4 ± 0.2	
	201.7	39.80 ± 0.1	15.7 ± 0.2	
Chitosan-starch composite film	LMW	5.77 ± 0.62	9.04 ± 1.42	[104]
	MMW	20.90 ± 3.52	4.67 ± 0.58	
	HMW	22.30 ± 2.21	9.09 ± 0.42	

#### 2.2.3. Solubility

The solubility of chitosan is a fundamental property that is particularly important in the fabrication of chitosan-based materials [17,26,105]. The main factors that affect this property are DD and MW. It is known that due to the high degree of acetylation, chitin is hydrophobic making it insoluble in water and most organic solvents, decreasing its applications [13]. On the other hand, with higher DD levels, more amino groups in the molecular chain become protonated to give higher degrees of solubility [106,107]. However, an increase in the MW brings about an increase in the intra- and inter-molecular hydrogen bonds within the chains, giving rise to entanglement of the chains and a reduction in solubility [108].Chitosan is soluble in weak acids but insoluble above a pH of 7. The pH has a significant influence on the charged state and properties of chitosan due to the presence of the amino groups [74]. At low pH, the amino groups of chitosan are protonated and become positively charged which leads to a soluble cationic polyelectrolyte. However, as the pH increases to above 6, the amino groups of chitosan are deprotonated, the biopolymer loses its charge, and this gives an insoluble structure. The soluble-insoluble transition occurs at about a pH of 6.5 (p*K*a of the amino group). This characteristic makes chitosan a cationic polyelectrolyte (p*K*a ≈ 6.5), one of the few found in nature [19,27].

In addition to the properties mentioned previously, solubility depends also on the type of acid used [13]. Formic acid is one of the best solvents when aqueous solutions of chitosan are required and the formic acid concentrations can range from 0.2–100% [14]. Acetic acid (1%) has been the most used solvent for the solubilisation of chitosan [13]. However, acetic acid solutions with high concentrations and at elevated temperatures can give rise to the depolymerisation of chitosan [109]. Rinaudo et al. [109,110] observed that for acetic and hydrochloric acid, the chitosan solubility was entirely related to the pH and to the ionic strength, while Kurita et al. [111] verified that it was dependent on chain flexibility, degree of ionisation, crystallinity, solvation of the chain, and the presence of acetyl-glucosamine blocks. Shamov et al. [112] have observed that chitosan solubility is also influenced by interactions between the hydrocarbon chains of the carboxylic acids. There are many other factors that have vital effects on chitosan solubility. These factors can include alkali concentration, temperature, time of deacetylation, prior treatments applied to chitin isolation, particle size, etc. [113]. In addition, these studies also highlight that solubility in acidic solution imparts the chitosan with excellent gel-forming properties and can expand the potential applications of chitosan-composite materials.

#### 2.2.4. Surface Area and Particle Size

Chitosan surface area and particle size are important characteristics which are related to the porosity, pore volume and pore size distribution of the chitosan. Surface area and particle size are fundamental for adsorption applications, since accessible sites and a porous structure are required [114,115]. It is known that chitosan powders or flakes are non-porous materials which present a low surface area (lower than 10 m^2^ g^−1^) [23]. Thus, chemical and physical modifications of chitosan have been performed to increase the surface area and improve potential applications [17,27,58,59,116,117]. Phongying et al. [118] obtained chitosan directly from chitin and prepared chitosan nanoscaffolds in order to improve the surface area, particle size and pore volume. They verified that the surface area of their chitosan scaffolds (55.75 m^2^ g^−1^) was approximately seven times higher than the commercial chitosan flakes (7.70 m^2^ g^–1^). Moreover, the pore volume and pore size of the chitosan nanoscaffolds were higher. Esquerdo et al. [119] developed chitosan scaffolds and verified that the new material had a specific surface area, porosity and pore volume of 1135 m^2^ g^−1^, 92.2% and 0.0079 m^3^ kg^−1^, respectively. These values are higher that other chitosan-based materials, such as chitosan powders (surface area of 4.2 m^2^ g^−1^ and pore volume of 9.5×10^−6^ m^3^ kg^−1^) [120], chitosan flakes (surface area range of 4–6 m^2^ g^−1^), chitosan beads (surface area range of 30–40 m^2^ g^−1^) [121], chitosan hydrogel beads (porosity of 85%) [122], and chitosan–graphene mesostructures (surface area of 603.2 m^2^ g^−1^) [123]. These studies confirm that modification of chitosan leads to an improvement in the surface area and, consequently, in the porosity and pore volume.

Moreover, the particle size of the adsorbents has a significant effect on the final solute concentration, and hence on the overall performance of the adsorption process. Larger particle sizes reduce the uptake due to the lower specific surface area. Thus, an increase in surface area of adsorbent results in new active sites formed, thus allowing more binding of solute molecules [124]. Piccin et al. [120] investigated the effects of particle size, surface area and pore volume of chitosan on the adsorption of FD&C Red 40. The particle sizes used were 0.10, 0.18 and 0.26 mm, with surface areas of 4.2, 3.4 and 1.6 m^2^ g^−1^, respectively. The results showed that an increase in the surface area and a decrease in particle size doubled the adsorption capacity. Dotto et al. [57] evaluated chitin and chitosan as adsorbents for tartrazine dye. They verified that chitosan showed better adsorbent properties than chitin due to its higher deacetylation degree and higher surface area, pore volume and pore size. These characteristics are particularly important for adsorption applications because it provides access to large pollutant molecules, enabling them to reach the internal adsorption sites.

## 3. Chitosan Supports

Although chitosan is an effective adsorbent for a variety of pollutants (as illustrated in Section 2), it nevertheless suffers from poor mechanical properties and thermal stability combined with a relatively low surface area. Therefore, it is not surprising that it has been modified with a variety of other additives to form composites or hybrids. These additives include cellulose [125,126], starch [127], other biopolymers such as alginate [128], gelatin [129], clays, such as bentonite [130], zeolites [131], metal organic frameworks (MOFs) [132], conducting polymers, such as polypyrrole [133] and other polymeric systems comprising methacrylamide [134],polyacrylamide [135], polyurethane [136], poly(vinyl alcohol) [137] and lignosulfonate [138]. These additives are interesting because they can form interpenetrated polymers with chitosan.

### 3.1. Chitosan Combined with Carbon-Based Materials

**Figure 4:**
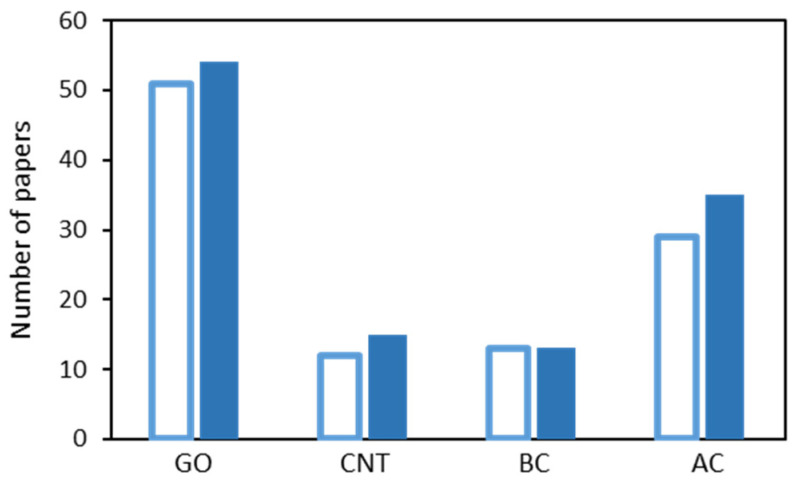
Number of papers published in 2019 (open) and 2020 (solid) focussed on various CS/carbon-based materials, where the carbon materials are graphene oxide (GO), carbon nanotubes (CNT), biochar (BC) and activated carbon (AC). All data taken from Scopus.

In more recent times, there has been considerable interest in combining chitosan (CS) with carbon-based materials as many carbon-based materials have very good adsorption qualities and these materials can also be employed to enhance the surface area of the adsorbents. Shown in Figure 4 is a summary of the number of papers published in 2019 and 2020 that have employed chitosan combined with various carbon-based materials as adsorbents. It is clearly evident from this analysis that it is graphene and especially graphene oxide (GO) that is dominating the carbon-based materials, with a somewhat lower number of papers describing the use of activated carbon. In the following sections, these CS/carbon-based materials are introduced, highlighting their properties and abilities to facilitate adsorption.

#### 3.1.1. Chitosan/Graphene Composites

Since its discovery, graphene has been used in a wide variety of applications, ranging from sensors [139], batteries [140], electro-Fenton [141] to electronics [142]. It has also been recognised as an adsorbent material, as it possesses a large surface area and there is considerable evidence to show that π-π interactions occur between the aromatic rings of various organic pollutants and the basal planes of graphene [143]. These π-π interactions occur between aromatic pollutants and pristine graphene, but fortunately graphene oxide, which is considerably easier to synthesise and is more cost effective, is an especially promising adsorbent [144]. GO contains a number of oxygen containing functional groups, such as epoxides (C–O–C), hydroxyl (–OH), carboxylic (–COOH) and carbonyl groups (>C=O) [145], while other oxygen containing groups, such as ketones and quinones, have also been detected [146]. These functional groups can facilitate the binding of positively charged molecules through electrostatic interactions [147]. Indeed, numerous studies have demonstrated the excellent ability of GO to adsorb various planar aromatic molecules, such as dyes, through a combination of π–π stacking, electrostatic interaction and hydrogen bonding [148,149].

**Figure 5:**
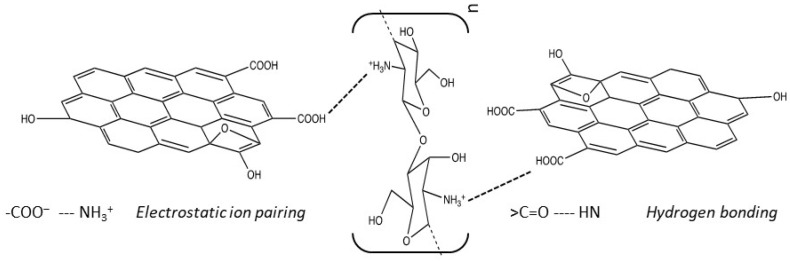
Schematic representation of the interactions between chitosan and GO.

Graphene oxide (GO) is normally synthesised by oxidising graphite using the well-known modified Hummers method [150]. The interlayer spacing increases as the graphite is oxidised to give GO sheets that can be exfoliated through a relatively simple liquid-phase exfoliation and/or ultrasonication. The GO sheets are stable in colloidal solutions and are easily combined with chitosan to give CS/GO composites. Typically, the chitosan is dissolved in acetic acid and the GO is added to form a homogeneous mixture. The CS/GO hydrogel can be easily formed, by a combination of violent shaking and sonication [144], adding NaOH [151], freeze drying [152], or by employing solvothermal reactions [153]. Chitosan is a positively charged polysaccharide at near neutral pH due to protonation of the amino groups and therefore it attracts the negatively charged GO sheets. These electrostatic interactions combined with hydrogen bonding facilitates the formation of the CS/GO hydrogel to give stable composites with excellent thermal and mechanical properties [154], as illustrated in the schematic provided in Figure 5. Indeed, it has been shown by Fan et al. [155] using FTIR measurements, that the –NH groups on the chitosan chains react with the –COOH groups of GO to form a linking –NHCO– group. Using these approaches, CS/GO composites have been formed as beads [156], membranes [157,158] and columns [144,159] and employed successfully as adsorbents for the removal of pollutants from aqueous media.

Several studies have been reported using CS/GO composites and these hydrogels have been employed to adsorb and remove various dyes from water [160], heavy metal ions [161], phenolic compounds [162] and pharmaceutical and personal care products [163]. In more recent years, other components have been added in an attempt to further enhance the adsorption capacity of the CS/GO composites, while three-dimensional GO and graphene based aerogels have also been developed and these are now described in turn.

##### Magnetic Chitosan/GO

Magnetic chitosan has emerged as an exciting new material in environmental applications and recently there has been much interest in the applications of magnetic CS/GO [164,165]. The introduction of magnetism facilitates the separation of the adsorbent from the aqueous medium through a simple magnetic process [166]. It is normally difficult to separate chitosan-based adsorbents, and indeed other adsorbents, from aqueous environments through conventional filtration and sedimentation techniques, as these adsorbents can block filters and are often lost, contributing to secondary pollution. The Fe_3_O_4_, a ferromagnetic black iron oxide, is the most widely employed, as it possesses good compatibility, low toxicity and also has high magnetic properties [167]. Furthermore, it contains both Fe(II) and Fe(III), and with the presence of Fe(II), which has the potential to act as an electron donor, oxidation of the pollutants can be achieved. Fe_3_O_4_ can also be formed as rods, spheres, wires and nanoparticles and these can be combined with CS/GO. There has also been a report where FeO(OH) was utilised with CS/GO [168], while γ-Fe_2_O_3_ has been combined with chitosan and employed as a magnetic adsorbent [169].

**Figure 6:**
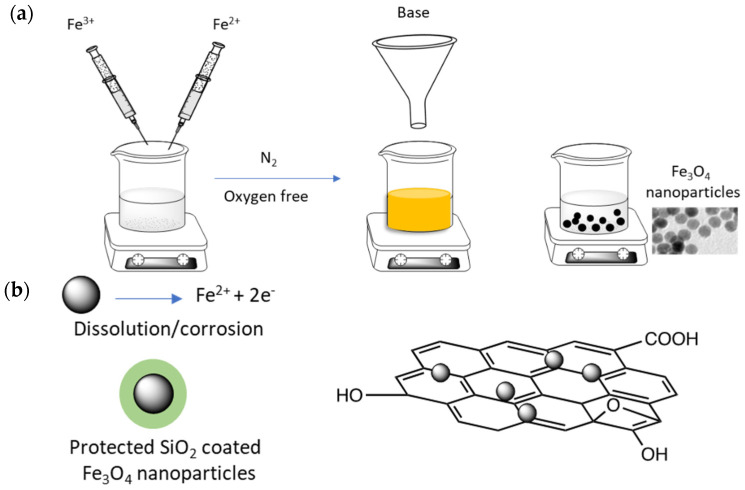
(**a**) Schematic representation of the co-precipitation method used to prepare Fe_3_O_4_ (**b**) SiO_2_ coated Fe_3_O_4_ nanoparticles deposited and dispersed on GO.

Magnetic CS/GO can be easily formed through both in-situ [170] and ex-situ methods [171] and variations of these two approaches. The GO/Fe_3_O_4_ can be initially formed before being combined with chitosan [172], or the CS/Fe_3_O_4_ can be firstly formed [173]. For example, Singh et al. [165] used the reactions between the carboxyl and epoxy groups on GO and the amine groups on chitosan to form amide and hydroxyl functionalised groups that facilitated the conversion of the iron ions to the iron oxide, enabling the in-situ preparation of the magnetic CS/GO. Alternatively, the Fe_3_O_4_ nanoparticles can be initially synthesised using simple methods, such as co-precipitation using ferric and ferrous salts, as illustrated in the schematic provided in Figure 6a. The Fe_3_O_4_ nanoparticles are then combined with the CS/GO hydrogel [174]. Using these approaches, Tran et al. [175] showed that a large number of the Fe_3_O_4_ nanoparticles were immobilised onto the GO sheets, Figure 6b, while Rebekah et al. [164] also concluded that the Fe_3_O_4_ nanoparticles became attached to the edges and basal planes of GO.

The dispersion and aggregation, size dispersion and shape of the Fe_3_O_4_ nanoparticles within the hydrogels are all important characteristics in terms of their performance as adsorbents. In general, the Fe_3_O_4_ nanoparticles appear aggregated, due to their magnetic nature [173]. Some authors have estimated the particle sizes or have observed some isolated particles among the clusters. Spherical Fe_3_O_4_ clustered particles were observed by Gul et al. [167] with some isolated particles of approximately 90 nm. Shafaati et al. [176] have prepared spherical Fe_3_O_4_ particles with an average size of 45 nm with evidence of some agglomeration, but when they were combined with chitosan an increase in the particle size was observed, indicating more extensive agglomeration during the reaction with chitosan or as the authors suggested, the chitosan polymer chains may provide links between the neighbouring Fe_3_O_4_ particles. Again, Jiang et al. [177] have shown that the Fe_3_O_4_ particles can become severely aggregated, but when the Fe_3_O_4_ particles were coated with silica the aggregation was markedly reduced. TEM micrographs indicated that the GO sheets were decorated with the silica coated Fe_3_O_4_ particles with the more wrinkled GO sheets providing more adsorption sites for the particles. A similar finding, highlighting the role of silica in reducing aggregation of the Fe_3_O_4_ particles, was reported by Tang et al. [178]. This reduction in the aggregation was attributed to a decrease in the dipole-dipole interactions between the silica modified Fe_3_O_4_ nanoparticles. Furthermore, the inert silica coating layers can protect the magnetic cores as the Fe_3_O_4_ particles are susceptible to dissolution and corrosion in acidic solutions, which lead to the loss of magnetism [179], as illustrated in Figure 6b.

##### Chitosan/rGO

While GO is the main form of graphene employed with chitosan, there is also evidence to show that reduced GO, designated as rGO, can be employed to give CS/rGO hydrogel adsorbent materials. The rGO is formed through the reduction of GO and this can be achieved using various thermal approaches, where the GO is heated to high temperatures to transform the oxygen-containing groups to gaseous CO or CO_2_ [180], reducing agents, such as borohydride or ascorbic acid [181], or through the electrochemical reduction of GO [182,183,184]. However, it is very difficult to completely reduce GO and maintain it in the fully reduced form and therefore rGO will always contain some oxygen-containing functional groups. The rGO is considerably more conducting compared to GO, and therefore it can be easily decorated with various metals or metal oxide particles or single atoms. Indeed, Pradeep and co-workers [185] employed the conducting nature and properties of rGO to form well dispersed and uncapped silver, gold, platinum, palladium and manganese oxide decorated rGO, which was then supported within a chitosan hydrogel. The redox reaction between the metal ion precursors and rGO leads to the progressive oxidation of rGO back to GO, providing the metal decorated graphene sheets with functional groups, facilitating its incorporation within chitosan.

CS/rGO has also been combined with Fe_3_O_4_ to give magnetic CS/rGO adsorbents and employed to give the effective adsorption of an antibiotic [186] and dyes [187]. While the conducting rGO can be beneficial in depositing well dispersed metal/metal oxide particles through reduction, there is evidence to show that CS/GO composites have a higher adsorption capacity when compared with the reduced GO counterparts. For example, Gu et al. [188] compared the performance of chitosan combined with GO and rGO in adsorbing and removing a dye from aqueous solutions and found that while adsorption was evident with both systems, the CS/GO was the more efficient adsorbent. This appears to be related to the presence of the functional groups providing a combination of π–π stacking, electrostatic interaction and hydrogen bonding with the pollutants [148,149].

##### Chitosan with 3D Graphene, Graphene Aerogels, Foams and Sponges

Although GO sheets can be well dispersed within chitosan, restacking of these sheets can occur over time to give GO aggregates and this, in turn, will reduce the surface area of the adsorbent, reducing its adsorption capacity. Consequently, there has been increasing interest in using three-dimensional (3D) GO or rGO hierarchical macrostructures for environmental applications [189,190]. The 3D GO structures can be fabricated as foams, sponges and as porous or macro-porous aerogels [191] and are based on the bending and wrinkling of the GO sheets to give a low mass density and very high specific surface areas [192]. These 3D materials have the potential to act as scaffolds with very good mechanical strength and a high specific surface area, facilitating adsorption. Moreover, they are easily recovered from the liquid phase following adsorption. However, 3D GO and rGO structures without any other additives can have relatively poor stability in water, but this stability can be enhanced considerably by combining the 3D GO network with biopolymers such as chitosan. Indeed, it was shown by Ma et al. [193], in studying the adsorption and removal of methylene blue, that the GO foam was susceptible to collapse, but its macroscopic morphology could be maintained over three repeated uses when combined with chitin. Similarly, 3D GO combined with high molecular weight chitosan was successfully applied in five repeated cycles of adsorption followed by regeneration, achieving a 90% adsorption capacity [152]. Very good stability and recyclability was also achieved with layered chitosan/GO sponges, with a regeneration efficiency greater than 80% over five cycles [194]. A number of CS/aerogel composites have been formed and these have been employed successfully in the removal of Cu(II) [195], tetracycline [196], azo dyes [197], anionic and cationic dyes [198], hexavalent chromium [199] and 4-nonylphenol [151].

##### Chitosan/GO with Other Additives

Other additives have been combined with CS/GO adsorbents and these have included β-cyclodextrins exploiting the hydrophobic properties of the β-cyclodextrin to enhance the adsorption of dyes. These β-cyclodextrin modified CS/GO composite materials have been fabricated and employed to adsorb methylene blue [200]. In this case the authors clearly showed that the extent of adsorption was enhanced on going from GO to CS/GO to CS/GO/β-cyclodextrin, illustrating the beneficial effects of incorporating the β-cyclodextrin. Yan et al. [201] employed a similar CS/GO/β-cyclodextrin composite to adsorb Mn(II), while Li et al. [202] found that the added β-cyclodextrin improved the adsorption of Cr(VI). Similar findings were reported in studying the adsorption of hydroquinone [203] and dye molecules [204].

Polypyrrole, a well-known conducting polymer, has also been combined with CS/GO by polymerising the corresponding pyrrole monomer within the CS/GO dispersion. This gives ternary hydrogel composites with a conducting polymer that has the ability to bind anionic and cationic species as dopants and these materials have been shown to give efficient adsorbents [205,206]. Moreover these hybrids can be further decorated with magnetic nanoparticles, enabling the removal of the adsorbent from water following the adsorption process [207]. Other polymeric systems that have been combined with CS/GO include polyacrylamide [208] and polyacrylate [209]. These high molecular weight polymers can improve the swelling and adsorption behaviour of the CS/GO hydrogels.

Although chitosan has a number of binding sites for metal ions, some of these are consumed in the crosslinking with the GO sheets. Consequently, additives that have additional binding sites have been added with the aim of enhancing the adsorption capacity. Particularly interesting additives include polydopamine, a mussel adhesive, that is easily formed through the oxidation and polymerisation of dopamine in slightly alkaline solutions [210]. It is a promising adsorbent material [211]. Polydopamine has a high density of amine and catechol groups and the combination of chitosan and polydopamine gives more binding groups and has been used to adsorb Cr(VI) [212] and Cu(II), Pb(II) and Cd(II) [213]. Other interesting materials are layered double hydroxides (LDHs) that have the general formula [M^2+^_1__–x_M^3+^_x_(OH)_2_]^x+^[(A^n^^–^)_x/n_mH_2_O] where M^2+^ and M^3+^ are the divalent and trivalent cations, respectively, such as Fe^2+^ and Al^3+^, while A^n−1^ represents the intercalating anions. These layered materials have very good adsorption properties for metal ions and have been used extensively for the removal of heavy metal ions [214]. It is not surprising that LDHs have recently been combined with chitosan and GO to give efficient adsorbents with enhanced adsorption performances [215,216]. Recently, metal-organic frameworks (MOFs) have also been combined with CS/GO [217,218] to give good adsorption properties. MOFs have received considerable interest in environmental science and chemistry as these materials have high porosity and high specific surface areas, with tunable pore structures. Indeed they have been used for heavy metal adsorption and are attracting applications in wastewater treatment [219]. However, MOFs, which are typically powders, are difficult to separate from aqueous environments and this is limiting their environmental applications. The CS/GO hydrogel provides a matrix for encapsulating these powdered materials and as detailed earlier the GO sheets can be easily decorated with magnetic iron to introduce magnetic separation.

A number of other additives has been combined with CS/GO, such as kaolin as a filler to enhance the mechanical strength of the hydrogel composite [220], lignosulfonate for additional binding sites [221,222], triethylenetetramine providing amine groups to enhance adsorption [223], hydroxyapatite to enhance strength and adsorption capacity [224] and silica as it contains a number of silanol groups (Si–OH) [225] and it can be furthermore employed to aid the dispersion of GO within chitosan to give effective adsorbents [226]. Moreover, other biopolymers have been combined with chitosan to form blends which are then combined with GO to give high performing adsorbents. These comprise CS/GO/gelatin [227], CS/GO/alginate [228], CS/GO/heparin [229] and CS/GO/cellulose blends [230].

#### 3.1.2. Chitosan/Carbon Nanotubes

Carbon nanotubes (CNTs), like GO sheets, have high surface areas and excellent stability. Therefore, there has been considerable interest in combining these carbon-based materials with chitosan to give adsorbent materials. CNTs are now readily synthesised as single-walled (SWCNT) and multi-walled nanotubes (MWCNT), distinguished by the number or graphitic layers folded over to form the tubes, with very high aspect ratios. They can be well dispersed within chitosan minimising their agglomeration. For good dispersion, the CNTs are normally treated in nitric acid to generate –COOH groups [231] and these groups can also bind with the chitosan. More recently, the CNTs have been functionalised with valine and starch to aid their dispersion within chitosan and enhance their affinity for the adsorption of heavy metal ions [232]. In addition, they have been coated with polydopamine thin films to aid dispersion and minimise aggregation within chitosan [233]. Similar to that employed in the formation of CS/GO, the CS/CNTs are formed by initially dissolving the chitosan in acetic acid, then the CNTs are added, dispersed and normally a crosslinking agent, such as glutaraldehyde [215], is used. These CS/CNT composites have been employed as adsorbents and used in the removal of Cr(VI) [234], V(V), Cr(VI), Cu(II), As(V) and Ag(I) from biological and environmental samples [235], Cu(II) [236], U(VI) [237], Pb(II) [238], phosphate [239], phenol [60], fluoride [58], diazinon [240], food dyes [241] and dyes [242].

Magnetic separation has also been developed and this provides a convenient method to remove the CS/CNT adsorbents from the aquatic environment. This is especially important for CNTs as there is considerable concern over the environmental and ecological risks associate with the release of CNTs into the environment [243,244]. For example, Zhou et al. [245] decorated CNTs with –NH_2_ functionalised super paramagnetic CoFe_2_O_4_ nanoparticles and combined these magnetic CNTs with chitosan and employed the resulting composites for the removal of Pb(II) and tetrabromobisphenol A. Magnetic Fe_3_O_4_ nanoparticles have also been used to form magnetic CS/CNTs composites and employed to remove Pb(II) [246].

Multicomponent and multifunctional CS/CNTs have also been formed. For example, CS/CNT has been further modified with poly(acrylic acid) and poly(4–aminodiphenylamine). The resulting adsorbent enabled the removal of Cr(VI) through adsorption and reduction to the Cr(III) species. The partially oxidised poly(4–aminodiphenylamine) was transformed in the presence of Cr(VI) into its fully oxidised form with the corresponding reduction of Cr(VI) to Cr(III) [247]. Alsabagh et al. [248] have fabricated a multifunctional nanocomposite comprising chitosan, well dispersed silver and copper nanoparticles and CNTs for the adsorption of Cu(II), Cd(II) and Pb(II). Other components have been added to CS/CNT and these include a prussian blue analogue [249], while a cellulose acetate (CA) and chitosan solution were used as an electrospinning solution and employed to form multicomponent electrospun CA/CS/CNTs/Fe_3_O_4_/TiO_2_ nanofibers [250].

CS/CNTs have also been formulated to give selective adsorption. While many adsorbents can give relatively high adsorption capacity, it is more challenging to obtain selective adsorption. One avenue that can be employed is imprinting technology. This has been used successfully with ion imprinted polymers, whereby the polymer is formed with a template molecule through a copolymerisation process. The template molecule is then removed leaving behind cavities in the polymer matrix with an affinity for that template, facilitating its rebinding. Li et al. [251] have used this approach to form CS/CNTs composites for the selective capture of Gd(III) by imprinting the chitosan with the Gd(III).

#### 3.1.3. Chitosan/Biochar

Biochar (BC) is a porous carbon rich material which is obtained through the pyrolysis of organic matter, in the presence of a limited concentration of oxygen. It has attracted much attention in environmental applications as it has a porous structure [252,253]. Moreover, it is a cost-effective material as it is fabricated from wastes, mainly agricultural and forestry waste materials. However, the adsorption capacity of biochar is limited and the density of the functional groups on its surface depend on the pyrolytic temperature with a general loss in these functional groups as the pyrolytic temperature is increased [253]. Accordingly, much attention has been paid to the modification of the biochar production process and modification of the surface through oxidation and/or functionalisation, to give more effective adsorbents [254,255]. Treatment of the BC with H_2_O_2_ is an interesting modification that gives rise to an increase in the concentration of the oxygen-containing functional groups and aids the removal of heavy metal ions from water [254].

Chitosan has been coated onto biochar surfaces [256] and employed as a dispersing and stabilising reagent to form CS/BC composites [257,258]. The BC powders are difficult to retrieve from aqueous solutions, but when the BC is incorporated within the chitosan hydrogel, it is more readily separated from the solution phase. Separation can be further facilitated by forming magnetic CS/BC hydrogels [259,260]. In addition, by using the chitosan solution phase, it is possible to add a number of other additives or reagents in addition to the BC, giving the composite more functional properties. For example, while CS/BC composites have a number of functional groups, such as amine and hydroxyl groups, other additives that increase the number of functional groups can be introduced within the hydrogel matrix. Using this approach, pyromellitic dianhydride (PMDA) has been employed as it can react with the amine groups of chitosan to give additional amides and carboxyl groups and this facilitates electrostatic interactions and complexation with heavy metal ions [261]. Indeed, it was found that the CS/PMDA modified BC exhibited selective adsorption for Cu(II) and this was attributed to the *N*-containing functional groups and carbonyl groups. Moreover, poly(acrylic acid), with carboxylate groups, was grafted to the chitosan modified BC to give not only additional functional groups, but also enhance chemical stability with stronger intermolecular forces [262]. Supramolecules, such as cyclodextrins, which have hydrophobic cavities and hydrophilic exteriors, and can form inclusion complexes with a wide range of organic molecules, have also been combined with CS/BC to give higher performing adsorbents [263]. These CS/BC composites have been employed as adsorbents in a number of studies to remove heavy metal ions from water [264], including Cr(VI) [265]. In addition, they have been utilised in the removal of phosphates [266], nitrates and phosphates [267], fluorides [268], benzoates [269] and various antibiotic and pharmaceutical molecules, such as diclofenac, ibuprofen and naproxen [270], ciprofloxacin [271,272] and ofloxacin [273].

#### 3.1.4. Chitosan/Activated Carbon

Activated carbon (AC) is well-known as an adsorbent material. It has been used in a number of environmental applications [274]. However, its relatively high cost is limiting its more widespread applications. One of the more commonly used starting materials in the synthesis of AC is coal [275], but given the depleted amounts of coal now available, this gives rise to an increase in the price of coal-based AC. Consequently, there is a recent focus on developing more environmentally acceptable synthesis and fabrication methods, using starting materials such as mandarin peel [276] and coconut shell [274]. Another avenue being exploited is the fabrication of multifunctional adsorbent materials that contain relatively small amounts of AC. Chitosan, with its high density of functional groups and good dispersion properties, is an ideal companion material. Indeed, there is evidence to suggest that this combination is effective as an adsorbent material. On comparing the maximum adsorption capacities of AC, chitosan and CS/AC for Cd(II), Hydari et al. [277] observed values of 10.3, 10.0, and 52.63 mg g^−1^ for AC, chitosan and CS/AC, respectively. Likewise, Auta and Hameed [278] observed synergistic effects between AC and chitosan in the removal of cationic and anionic dyes, while Fatombi et al. [279] also concluded that the best performance was achieved with a CS/AC composite.

CS/AC composites have been formed using commercially available activated carbon, coconut shell charcoal/carbon [280,281], renewable waste tea [278], sapotaceae seed shells [282] *Typhalatifolia* leaves [283] and olive stones as the carbon source [284]. These composites can be formed through a surface modification process, where the surface of the AC is modified by chitosan [285]. Babel et al. [280] concluded that surface modification of coconut shell charcoal with chitosan significantly improved the adsorption of Cr(VI). They also found that the pre-treatment of the AC with acids gave rise to enhanced adsorption. Amuda et al. [281] arrived at a similar conclusion, and showed that chitosan coated acid treated coconut shell carbon was very effective in the removal of Zn(II). Alternatively, the chitosan can be dissolved in acid and then the AC can be added in the form of a powder to generate CS/AC [286,287]. Crosslinking agents, such as glutaraldehyde [288], genipin [289] or epichlorohydrin [290], can be added to generate the composite hydrogels. With this latter approach, the ratio of activated carbon to chitosan can be easily varied [291], while other additives can be introduced. For example, a CS/AC was formed with SiO_2_/Fe_3_O_4_ to give magnetic CS/AC [288], while CS/AC was combined with an anionic surfactant, sodium dodecyl sulphate (SDS), to adsorb a cationic dye [292]. In addition, chitosan has been blended with polyvinyl alcohol [293,294], and polyethylene glycol [295] and then combined with activated carbon, while CS/CA has also been combined with alginate to form CS/CA/alginate adsorbent beads [289].

Activated carbon fibres, regarded as the third generation of carbonaceous adsorbents, have also been employed with chitosan. These have been utilised as membranes [296] and have been decorated with iron oxides and modified with chitosan to remove arsenic, phenol and humic acid from water, with high adsorption capacity for As(V) [297]. Magnetic activated carbon nanofibers based on chitosan and cellulose acetate have also been fabricated for the adsorption of Cr(VI), Ni(II) and phenol from aqueous solutions [298]. In addition, a number of magnetic CS/AC composites has been formed with the majority involving Fe_3_O_4_ [299,300], while others have employed CoFe_2_O_4_ [301] and barium ferrite [302].

These CS/AC composites have been evaluated for the removal of phenols [303], parabens [304], dyes [305], food dyes [306], anti-inflammatory drugs [307], acetaminophen [308], organic molecules, such as aniline [309], and various heavy metal ions [289]. Generally, there is good agreement that the combination of chitosan and AC gives rise to enhanced adsorption, when compared to the individual chitosan and AC counterparts.

### 3.2. Chitosan Combined with Inorganic Adsorbent Materials

While chitosan has been combined with various carbon based materials, as illustrated in Section 3.1, there is growing interest in the use of inorganic components, such as activated alumina [310], mesoporous alumina [311], silica and ordered mesoporous silica-based materials, as the chitosan support materials [312,313]. Silica has very good physical, mechanical and thermal stability and can be easily functionalised due to its hydroxyl groups. In particular, mesoporous silica is a fascinating material, which first gained prominence in the 1990s with a regular mesostructure, with uniform pore distribution and tunable pore sizes, very high specific surface areas, combined with thermal and mechanical stability [314]. It is attracting considerable interest as an adsorbent material [315]. These materials can be formed by a simple sol–gel synthesis route comprising hydrolysis, condensation and polycondensation reactions using various templates or surfactant molecules [316]. In particular, the template-assisted mesoporous silica synthesis using surfactants is gaining considerable attention. Typically, liquid silicon alkoxide precursors, such as tetramethyoxysilane or tetraethoxysilane are used. The successive polymerisation, gelation, drying and aging steps can be tailored to control the microstructure of the final materials. The surfactant–silica assembly occurs simultaneously with condensation of the inorganic species to produce the mesoporous silica composite.

CS/silica composites have been formed using a variety of methods which can be broadly grouped into two main approaches, comprising silica supported chitosan, where the chitosan is coated or adsorbed onto the silica support, and secondly a CS/silica hybrid that is fabricated using the sol-gel methodology. Several reports have focussed on SiO_2_ as a bead, particle, nanoparticle or powder, where the SiO_2_ particles are added to the chitosan solution phase to give a chitosan coated particle [317]. The SiO_2_ particles can also be functionalised with amine and carboxylic groups to give more efficient binding with the chitosan [318]. Silica layers have also been added to previously formed chitosan-based beads to give organic-inorganic (CS/silica) layered structures, with greater stability [319] and sol-gel synthesis has been employed to immobilise chitosan onto silica particles [320]. Sol-gel synthesis is more commonly used to form a CS/silica hybrid layer on silica bead/particle supports [321,322]. For example, Xu et al. [323] covalently linked chitosan with an epoxide containing siloxane through the sol-gel process to give a hybrid chitosan layer on silica particles. The CS/silica hybrid has been further modified with EDTA (ethylenediaminetetraacetic acid), which is very well known to form stable chelates with a number of metal ions [324], to give adsorbents for heavy metal ions [325]. While the sol-gel synthesis is very versatile, Blachnio et al. [326], on comparing three CS/silica composites formed by the adsorption of chitosan on silica gel and fumed silica and by the sol-gel process, concluded that the adsorbed chitosan had a higher adsorption capacity for dye molecules, although the CS/silica fabricated using the sol-gel synthesis had a high surface area of 600 m^2^ g^−1^.

There has been considerable interest in combining mesoporous silica with chitosan to combine the good adsorption properties of chitosan with the large surface area and adjustable pore size of silica. Likewise, magnetic mesoporous silica, which has a magnetic Fe_3_O_4_ core surrounded by the mesoporous silica is attracting a lot of attention in environmental applications [327]. These magnetic materials are environmentally acceptable with no toxicity, are biocompatible, have high surface area, very good stability and the outer mesoporous silica can be functionalised and modified by chitosan to add functional groups. The cross-linking method can be employed to decorate the mesoporous silica with the chitosan. Cross-linking agents, such as glutaraldehyde [328], formaldehyde [329] and epoxides [327], can be used, while in a recent study, He et al. [330] used thiol-ene click chemistry to achieve binding between chitosan and magnetic mesoporous silica. The surface areas, pore sizes and volumes of a number of these materials are summarised in Table 4. In general, the surface area, pore size and volume of the mesoporous silica are reduced as higher amounts of chitosan are added and partially fill the pores. However, these chitosan and mesoporous silica composites possess good surface areas with a high density of functional groups and with the potential to give magnetic separation.

## 4. Adsorption and Removal of Pollutants

The removal of pollutants from aquatic environments through adsorption is one of the more popular approaches in environmental applications. The aim in these technologies is to remove the maximum amount of pollutant and therefore adsorption isotherms have been used extensively to develop an understanding of the adsorption equilibria. In this section, these adsorption models are briefly introduced, followed by adsorption kinetics and finally a comparison of the performance of the various chitosan composites is made.

### 4.1. Adsorption Models and Adsorption Kinetics

**Figure 7:**
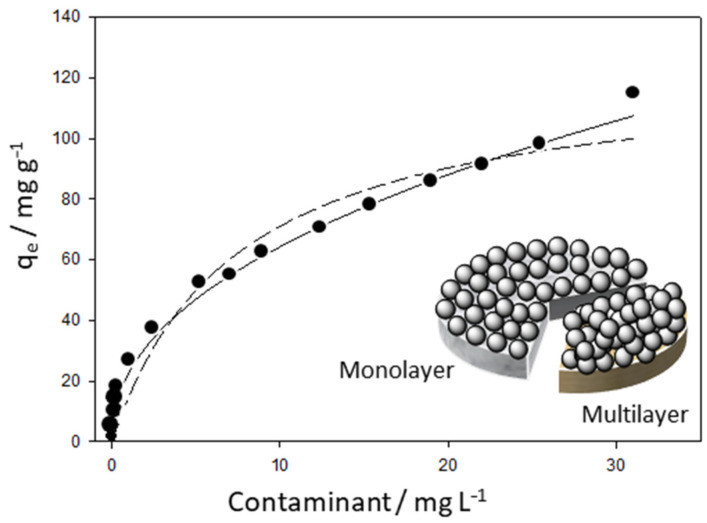
Schematic of an adsorption plot showing experimental data (symbols), with the –––Freundlich isotherm and − − − Langmuir isotherm fitting and the inset shows monolayer and multilayer adsorption processes.

Adsorption isotherms are frequently employed in the study of adsorption, facilitating a quantitative comparison of different adsorbent materials. In addition, they are often used to optimise the use of adsorbents, by observing the adsorption capacity as a function of the experimental conditions. Several different isotherm models have been employed to analyse experimental adsorption data and these include the Langmuir, Freundlich, Temkin, Frumkin, Redlich-Peterson (R-P), Halsey, Henderson and Dubinin-Radushkevich isotherms. However, the two most frequently used models with chitosan and chitosan-based composite materials are the Langmuir [223,334] and to a lesser extent the Freundlich isotherms [335]. The Langmuir adsorption model is described in Equation (1) and the linear form commonly employed in fitting data in Equation (2). Here *q_e_* is the equilibrium concentration of the adsorbate, *q_m_* is the monolayer adsorption capacity, *C_e_* is the concentration of the adsorbate in the aqueous phase and *K* is a constant. In this model all sites are considered as energetically equivalent, to give monolayer adsorption with no interactions between adjacent adsorbates. In this analysis, the adsorbent has a finite capacity for the adsorbate and a saturation point is reached where no further adsorption occurs. The BiLangmuir model can also be applied with chitosan-based composites [317] and, in this case, the relationship is given in Equation (3), where *q*_*m*1_ and *q*_*m*2_ represent the maximum adsorption capacities of two different adsorption sites and *K*_*L*1_ and *K*_*L*2_ correspond to these two sites. The Freundlich model assumes multilayer adsorption on a heterogeneous surface and can be described by Equations (4) and (5), where *q_e_* represents the amount of adsorbent adsorbed at the surface, *C_e_* is the equilibrium concentration, and *n* and *K_F_* are the Freundlich constant and Freundlich exponent, respectively. The Freundlich constant, *K_F_*, provides a measure of the adsorption capacity and the magnitude of *n* is related to the extent of adsorption with *n* > 1, indicating favourable adsorption. An adsorption plot, using simulated data, is illustrated in Figure 7, where a schematic of monolayer and multilayer adsorption is also shown. In this example, the experimental data are more consistent with the Freundlich isotherm.

(1) $$q_{e}=\frac{q_{m}K_{L}C_{e}}{1+K_{L}C_{e}}$$

(2) $$\frac{C_{e}}{q_{e}}=\frac{1}{K_{L}q_{m}}+\frac{C_{e}}{q_{m}}$$

(3) $$q_{e}=\frac{q_{m1}K_{L1}C_{e}}{1+K_{L1}C_{e}}+\frac{q_{m2}K_{L2}C_{e}}{1+K_{L2}C_{e}}$$

(4) $$q_{e}=K_{F}C_{e}^{1/n}$$

(5) $$\log q_{e}=\log K_{F}+\frac{1}{n}\log C_{e}$$

The kinetics of the adsorption process are important as these studies provide information on the rate of adsorption which is relevant in terms of the contact time required to remove the maximum amount of adsorbate. The Lagergren rate equation is one of the most widely used adsorption rate equations for the adsorption of adsorbates from a solution phase and this has been used with various chitosan composites [336]. The pseudo-first order (PFO) and pseudo second-order models (PSO) are described in Equations (6) and (7) where *q_t_* and *q_e_* represent the mass of the adsorbing molecule per unit mass of adsorbent at time *t* and at equilibrium, while *k*_1_ and *k*_2_ correspond to the first- and second-order rate constants and *t* is the time. Other kinetic models have been employed in the study of chitosan composite materials and these include a double-exponential kinetic model [337], and a generalised fractal kinetic model (Brouers-Sotolongo model) [338]. Generally, the adsorption kinetics are controlled by the (i) rate of diffusion of the adsorbate from the bulk solution to the adsorbent-solution boundary, (ii) diffusion form the boundary layer to the adsorbent surface, (iii) diffusion of the adsorbate within the adsorbent material, i.e., intraparticle diffusion and (iv) the rate of the adsorption step. Normally, the diffusion process in the bulk solution can be eliminated through agitation, while the adsorption is fast and the rate-determining step is typically intraparticle diffusion [200].

(6) $$\ln\left(q_{e}-q_{t}\right)=lnq_{e}-k_{1}t$$

(7) $$\frac{t}{q_{t}}=\frac{1}{q_{t}}+\frac{1}{k_{2}q_{e}^{2}}$$

### 4.2. A Comparison of the Chitosan Supported Composites in the Adsorption of Pollutants

The performance of the chitosan composites in the removal of heavy metal ions is summarised and illustrated in Table 5, Table 6 and Table 7, where the chitosan is combined with GO, CNTs, BC, AC and silica as support materials.

The Langmuir isotherm is based on the assumption that the surface of the absorbent is homogenous, and every adsorption site is equal. Although these chitosan-composites have various functional groups, which in turn, give rise to different affinities with the adsorbates, the Langmuir model and to a lesser extent, the Freundlich model, correlate well with most of the experimental studies. It is also evident in Table 5, Table 6 and Table 7, that many of the 3D or porous supports give higher adsorption capacity values, highlighting the influence of the more porous materials, while the magnetic chitosan materials, (MSC), with Fe_3_O_4_ particles/nanoparticles, also perform well. It is difficult to make a direct comparison between the carbon-based materials and silica-based supports, as the adsorption capacity depends on the nature of the chitosan. However, some of the highest adsorption capacities are seen when chitosan is combined with GO.

It can be seen from these tables that a large variety of heavy metal ions have been adsorbed and removed. This is not surprising as these ions are toxic and pose a significant threat to both human beings and aquatic life. As illustrated, the adsorption capacity varies considerably from relatively low values of 9.4 mg g^−1^ to much higher values in the vicinity of 957 mg g^−1^. These variations appear to be somewhat related to the nature of the heavy metal ions, with relatively high adsorption values for Pb(II) and lower values for Cr(VI). However, the nature and properties of the chitosan, including its DD levels, MW, porosity, particle size, solubility (Section 2), crosslinking agents and the ratio of chitosan to the carbon-based or silica supports are also important elements that will influence the extent of adsorption.

**Table 7:** Adsorption performance of CS/silica composites in the removal heavy metal ions from water.

Adsorbent	Adsorbate	pH	Ads. Capacity /mg g^−1^	Kinetic Model	Isotherm Model	Ref
CS/Silica	V(V)	6.5	16	-	-	[329]
Mesoporous	Cu(II)	6.5	21	-	-	
	Pb(II)	6.5	22	-	-	
	Cd(II)	6.5	12	-	-	
	Hg(II)	6.5	13	-	-	
MCS/Silica/ PAM	Cu(II)	5.0	43	PSO	Langmuir	[356]
	Pb(II)	5.0	63	PSO	Langmuir	
	Hg(II)	5.0	263	PSO	Langmuir	
CS/SBA-15	Pb(II)	5.0	57	PSO	Langmuir	[357]
MCS/SBA-15	Cu(II)	6.0	107	PSO	Langmuir	[333]
	Zn(II)	6.0	100	PSO	Langmuir	
MCS/SBA-15	Zn(II)	6.0	107	PSO	Langmuir	[358]
CS/Silica	Hg(II)	6.0	204		Langmuir	[359]
	As(V)	6.0	198		Freundlich	
CS/KCC-1	Pb(II)	9.0	168	PSO	Langmuir	[360]
MSC/Silica	Cu(II)	5.0	73	PSO	Langmuir	[361]
SC/Silica	Cr(VI)	3.0	9	PSO	Langmuir	[362]
MSC/Silica	Cu(II)	6.0	350	-	Freundlich	[363]
MSC/Silica aerogel	Cd(II)	8.0	71	PSO	Langmuir	[364]
CS/GO/Silica	Pb(II)	6.0	256	PSO	Langmuir	[225]
MCS/Silica	Cr(III)	4.0	39	PSO	Bi-langmuir	[317]

Abbreviations: polyacrylamide (PAM).

**Figure 8:**
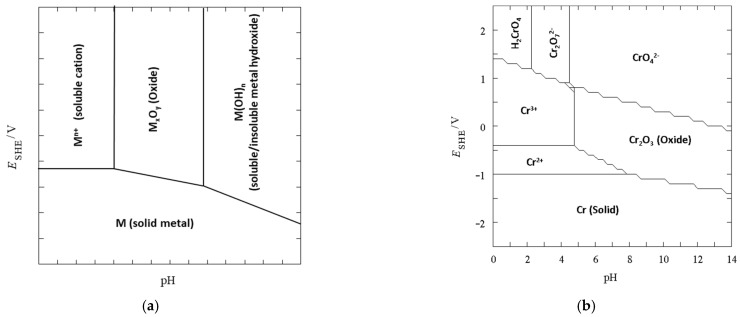
Pourbaix diagrams illustrating the speciation of (**a**) heavy metals, M, and (**b**) Cr in aqueous solutions.

In terms of the experimental conditions employed in these studies, it is well documented that the pH plays a significant role in the adsorption process. It is well known that the –NH_2_ and –OH groups on the chitosan chains have a strong association with metal cations and this facilitates the adsorption of various heavy metal ions at slightly acidic or near neutral pH values. This is clearly evident in Table 5, Table 6 and Table 7, where several of the studies are performed in slightly acidic solutions. As the chitosan becomes protonated, with the formation of NH_3_^+^ at lower pH values, this protonated group repels the cationic heavy metal ions. Moreover, the chitosan becomes less stable and more soluble in highly acidic environments. Indeed, the pH of zero charge (pH_pzc_) has been determined as 6.0 for CS/GO composites, indicating that the surface of CS/GO is positively charged for pH values < 6.0 but for pH values > 6.0, the surface adopts a negative charge [365]. However, the speciation of the metal ions is also important, and this is illustrated in Figure 8, where the Pourbaix diagrams for various heavy metals, designated as M, and Cr are shown. In general, for heavy metals, such as Cu(II), the adsorption capacity increases gradually as the pH increases from about 2 to 7 and then it decreases rapidly as the pH is further increased. The rapid decrease at higher pH values is due to the formation of insoluble hydroxide species. While higher concentrations of the metal cations are present in the solution phase at low pH values, the adsorption capacity is poor, which can be attributed to the protonation of chitosan. As the pH is increased, the–NH_2_ chelating groups become available and at these conditions the concentrations of the metal cations is still sufficiently high to facilitate chelation with the chitosan. The nature of the –COOH functional groups on GO and the other carbon-based materials is also pH dependent with the generation of –COO^−^ at higher pH values and again this anionic group will bind with metal cations. Therefore, the maximum adsorption is seen at pH values from about 5 to 7, as illustrated in Table 5, Table 6 and Table 7, for a number of heavy metal ions. The Pourbaix diagram of Cr is somewhat different with the generation of the anionic dichromate (Cr_2_O_7_^2−^) and chromate (HCrO_4_^−^) ions at the lower more acidic pH values and the insoluble oxide phases at higher pH values. In this case, the HCrO_4_^−^ ions can be adsorbed at the chitosan through electrostatic interactions with the protonated chitosan, to give more favourable adsorption at pH values between approximately 2.0 and 4.0.

Other conditions that can alter the adsorption capacity of the heavy metal ions are temperature and ionic strength. Generally, the adsorption capacity increases with higher temperatures [195], as Δ*G* becomes more negative, implying that the adsorption process becomes more favourable at higher temperatures, with Δ*G*° < 0, Δ*H*° > 0 and Δ*S*° > 0 for several adsorbents, however, adsorption can also be exothermic [337]. There have been relatively few studies devoted to the selectivity of the adsorption process at these chitosan composites. This is especially important in terms of the potential applications of the adsorbents, as the water samples or industrial effluents are likely to contain other cations and anions, such as Cl^−^, NO_3_^−^, SO_4_^2−^, Mg^2+^ and Na^+^. For example, it was found that an increase in the ionic strength inhibited the adsorption of Cu(II) at CS/GO [195]. One approach that can be employed to enhance the binding of a particular heavy metal ion is templating [251]. This is generally successful provided the target metal ion is reasonably different to the size of the co-existing ions.

The adsorption capacity of the various supported chitosan composites in the removal of dye molecules and other organic molecules, including antibiotics, is summarised in Table 8. Various cationic and anionic dyes have been employed as model compounds and very impressive adsorption capacities have been obtained in a number of studies. In particular, the adsorption of methylene blue (MB), a cationic dye molecule, is very high at CS/GO composites reaching values > 1000 mg g^−1^ in a number of studies, as illustrated in Table 8. This good adsorption is attributed mainly to the π−π interactions between the MB and GO layers. The electrostatic interactions between the –COO^−^ groups on GO and the cationic MB can also facilitate adsorption, provided the solution pH is not acidic giving rise to the formation of the unionised –COOH groups. Therefore, it is the GO that is largely responsible for the adsorption of cationic dyes [144]. On the other hand, the chitosan plays a more significant role in the adsorption of anionic dye molecules. Although π−π interactions will exist between GO and the anionic dyes, the electrostatic repulsion between the –COO^−^ groups and the anionic dye will inhibit its adsorption at higher pH values, where the COOH groups are ionised. The electrostatic interactions between the cationic centres in the chitosan chains and the anionic dyes will have a significant effect at low pH values, while a combination of electrostatic forces, van der Waals interactions and hydrogen bonding are likely to occur at higher pH values [366]. This explains the good adsorption of anionic dyes observed at near neutral pH values in Table 8. The concentrations of the dye molecules can also influence the adsorption process. Many of these dye molecules can form dimers and aggregates and this becomes more relevant as the concentrations of the dye molecules increase, with aggregates forming in solution and at the surface. Indeed, the impressive adsorption of rhodamine B [367] and its adsorption kinetics were questioned as the aggregation of rhodamine B in water was not taken into account in the original study [368].

Various antibiotics have also been adsorbed at the chitosan-based composites, as shown in Table 8. Again, many of these molecules have benzene rings which facilitate their adsorption onto the carbon surface through π-π electron donor-acceptor interactions. Furthermore, many of these organic molecules have –OH, >C=O and –NH_2_ groups which can be involved in hydrogen bonding with the oxygen groups on the carbon surfaces in the chitosan composites [369]. Hydrophobic interactions may also be relevant [370]. These organic molecules tend to be hydrophobic making hydrophobic interaction between the antibiotics and the carbon surfaces possible. However, high numbers of oxygen-containing groups, such as –OH and –COOH on the carbon surfaces tend to make the surface more hydrophilic.

The CS/silica composites are only emerging as potential adsorbents and compared with the chitosan-carbon based systems, there are much fewer reports focused on the removal of dyes and organic molecules with these adsorbents. This may be due in part to the silanol groups, which are hydrophilic, and easily form hydrogen bonds with water, thus limiting the adsorption process. However, the mesoporous silica surfaces can be functionalised, and this provides the opportunity to design more hydrophobic surfaces that can be tailored to adsorb organic molecules. Indeed, there is clear evidence in Table 8 that the CS/silica composites can be employed in the removal of dyes.

**Table 8:** Adsorption performance of chitosan composites in the removal pharmaceuticals, organics and drug molecules from water.

Adsorbent	Adsorbate	pH	Ads. Capacity	Kinetic Model	Isotherm Model	Ref
CS/GO	MB	5.3	95	PSO	Langmuir	[155]
MCS/GO β-CD	MB	-	84	PSO	Langmuir	[204]
CS/GO monoliths	MO	4.2	567	PSO	No fit	[371]
MCS/GO	Acid orange	3.0	42	PSO	Langmuir	[372]
MCS/GO	MO	4.0	398	PSO	Langmuir	[373]
CS/GO aerogel	MO	4.0	686	PSO	-	[197]
	Amido black	4.0	573	PSO	-	
CS/GOβ-CD	MB	12.0	1134	PSO	Freundlich	[200]
CS/GO aerogel	Metanil yellow	6.8	430	PSO	Langmuir	[365]
CS/GO aerogels	Indigo carmine	6.8	534	-	Langmuir	[198]
	MB	7.0	168	-	Langmuir	
CS/GO/Ligno-sulfonate aerogel	MB	7.0	1023	PSO	Langmuir	[221]
CS/GO/ Cellulose	MB	6.0	3190	PSO	Langmuir	[230]
MCS/GOCS-EDTA	Rhodamine B	7.5	1085	PSO	Langmuir	[367]
CS/GO aerogel	Congo red	7.0	384	PSO	Langmuir	[374]
MCS/GO	MB	8.5	2478	PFO	Sips	[170]
MCS/CNT	Congo red	6.0	262	PSO	Langmuir	[375]
MCS/CNT	Acid red	3.0	809	PSO	R−P. Freundlich	[376]
MSC/CNT/ SiO_2_	DB 71	6.8	61	PSO	Langmuir	[377]
	RB 19	2.0	97	PSO	Langmuir	
CS/CNT	DB 71	6.2	29	PSO	Langmuir	[378]
	FdR17	3.0	1508	Avrami	Langmuir	[241]
	FdB1	3.0	1480	Avrami	Langmuir	
CS/CNT/GO	Rhodamine B	-	9.6	PSO	-	[215]
CS/CNT	Phenol	6.5	404	PSO	Dubinin-Radushkevic	[60]
CS/AC	Crystal violet	9.0	12.5 (323K)	PSO	Langmuir and Freundlich	[283]
CS/AC	FBL2	3.0	155	Avrami	-	[286]
CS/AC	FR17	3.0	133	Avrami	-	
CS/AC/PVA	MB	6.0	468	PFO	Langmuir and Freundlich	[294]
CS/AC/PEG	MB	7.0	424	PSO	Langmuir	[295]
CS/AC mesoporous	Thionine	3–11	61	PSO	Freundlich	[305]
CS/AC	Indigo carmine	3.0	208	PSO	Langmuir and Freundlich	[279]
CS/AC with Carica papaya seeds	MB	8.0	302	PSO Elovich	Langmuir	[379]
CS/AC/hexa-decylamine	RB 5	4.0	666	PSO	Freundlich	[380]
CS/AC/ Cellulose	Tylosin	7.0	59	PSO	Langmuir	[381]
CS/Silica	Acid Red 88	7.0	25	PSO	Langmuir	[320]
MCS/Silica/ Glu	MB	5.0	185	PSO	Langmuir	[382]
	Crystal violet	5.0	390	PSO	Langmuir	
	Light yellow	5.0	228	PSO	Langmuir	
MCS/Silica	Rhodamine B	-	191	-	Langmuir	[383]
CS/Silica/ ZnO	MB	7.0	293	-	Langmuir	[384]
CS/Silica/ PVA	Direct Red 80	2.0	322	PSO	Langmuir	[385]
CS/GO	Tetracycline	9.0	1130	PFO	Temkin	[196]
MCS/GO	Rifampicin	5.0	102	PSO	Langmuir	[176]
CS/GO	Ciprofloxacin	7.0	5.3	-	Langmuir	[227]
	Ofloxacin	7.0	8.3	-	Langmuir	
MCS/rGO	Cefixime	6.4	30	-	Freundlich	[187]
CS/CNT	Diazinon	5.5	222	PSO	Sips	[240]
CS/BC beads	Ciprofloxacin	3.0	76	PSO	Langmuir	[386]
CS/BC	Ciprofloxacin	7.0	106	PSO	Langmuir	[272]
	Enrofloxacin	7.0	100	PSO	Langmuir	
MCS/AC	Ciprofloxacin	-	90	PSO	Langmuir	[299]
	Erythromycin	-	178	PSO	Langmuir	
	Amoxicillin	-	526	PSO	Langmuir	

## 5. Conclusions and Future Perspectives

It is clear from the reports reviewed and the growing number of publications, where chitosan and chitosan-based materials are employed as adsorbents, that these materials are emerging as interesting candidates in the formulation of adsorbents for environmental applications. Chitosan can be easily combined with different support materials, and while earlier studies were devoted to blending chitosan with other polymeric materials, many of the more recent reports are focused on combing chitosan with carbon-based materials with GO, and to a lesser extent activated carbon, attracting considerable attention. Likewise, there is increasing attention being focussed on merging chitosan and mesoporous silica. The CS/GO composites have shown impressive adsorption capacity with both dyes and heavy metal ions.

However, this research field is still in its infancy and a number of challenges exist and must be addressed before these chitosan-carbon based or chitosan-silica based materials can be employed as adsorbents for the removal of a variety of pollutants. One of the more challenging aspects, that has direct implications in terms of costs, is the regeneration of the adsorbents. Ideally, adsorbents should have the capacity to be regenerated and used multiple times. Regeneration is normally achieved using NaOH or acid treatments, where, for example, heavy metal ions are released from the chitosan. However, these treatments lead to a progressive hydrolysis of the polysaccharide on the chitosan. Consequently, the adsorption capacity decreases with each adsorption-regeneration cycle. New regeneration processes are required to give more longer lasting and cost-effective chitosan-based adsorbents. Other challenges are the introduction of selectivity in the adsorption process. Real water samples contain a number of ions that will compete with the removal of heavy metal ions, consuming the adsorption sites and reducing the uptake of the targeted pollutants. Furthermore, the removal of neutral pollutant molecules using these chitosan-based materials is more difficult to achieve, although the addition of GO provides two dimensional sheets that facilitate the adsorption of aromatic ring structures. In addition, the adsorbents need to be removed from the aquatic environment or employed in a continuous flow system. While the development of magnetic chitosan-based materials provides the opportunity to remove the adsorbents using magnetic separation, these magnetic materials are only emerging and it is not entirely clear if they can be sufficiently anchored within the chitosan composites to prevent their leaching over longer terms. However, the development of silica coated and protected magnetic iron-containing particles is promising. There are added concerns over the environmental impact of GO and CNTs, which if leached from the chitosan composites, can enter the aquatic system and have adverse effects on the aquatic ecosystem. Therefore, the CS-carbon and CS-silica composites must be stable and not prone to leaching of GO flakes, CNTs, or the magnetic iron oxide particles. Consequently, studies that monitor the leaching of the various carbon, silica and iron species from the chitosan composites are needed from an environmental perspective.

Fundamental studies on kinetics and intraparticle diffusion require further study. Most of the kinetic models employed are relatively simple pseudo-second order models while the impermeable nature of the GO sheets, CNTs, carbon and silica particles on the internal diffusion of the pollutants are not well developed. While the physical and chemical properties of chitosan can be tailored by varying its molecular weight, DD levels, particle sizes, etc, the complex relationships between some of these parameters and how they control the adsorption process and capacity are difficult to establish.

Nevertheless, these chitosan-based materials, and especially the emerging chitosan-carbon and chitosan-silica based composites, have a promising future as adsorbent materials. While chitosan is currently unable to compete with activated carbon in commercial and industrial settings, it is nevertheless an attractive and viable material as it is derived from chitin, which can be found in abundance and extracted from seafood wastes. With further developments aimed at strengthening the mechanical properties of chitosan, the development of recovery protocols, scale-up of production using green solvents and implementation of nonthermal technologies, industrial exploitation can become a reality. Moreover, there has been a recent explosion in the development of new two-dimensional materials. Some of these 2D layered materials, for example MXenes, MoS_2_ and MoSe_2_, are potential adsorbents and could be easily combined with chitosan to generate a new family of high-performance materials.

## Figures and Tables

**Table 1:** Main chitosan properties, according to the information reported in [13,27].

Physicochemical properties	Linear aminopolysaccharide with a high nitrogen content Ionic conductive Cationic biopolymer with high charge density Flocculating agent Complexing and chelating properties Existence of reactive groups for chemical activation and cross-linking Adsorption properties pKa varies from 6.5 and 6.7 Enable to form intermolecular hydrogen bonds
Biological properties	Bioadhesivity Non-toxic Bioactivity Adsorbable Biodegradable Antimicrobial activity Blood anticoagulants Antiacid, antiulcer and antitumoral properties Hypolipidermic activity

**Table 2:** Influence of deacetylation degree (DD) on physicochemical properties of chitosan in different forms.

Form	DD (%)	Properties	Results	Ref.
Films	82, 80–85, 100	Crystallinity, tensile strength, % elongation, swelling index	Crystallinity and tensile strength increased, while % elongation and swelling index decreased with increase in DD (highest MW)	[75]
Powder	76–92	Thermal degradation	Thermal stability decreased with increase in DD	[76]
Films	70–95	Crystallinity, tensile strength, % elongation, swelling index	Crystallinity and tensile strength increased with increase in DD, swelling index decreased with increase in DD; % elongation increased when the DD increased from 70% to 80% and decreased when the DD increased from 80% to 95%	[77]
Microspheres	48, 62, 75	Swelling index	Swelling index increased with increase in DD	[78]
Membranes	75, 87, 96	Crystallinity, swelling index, tensile strength, % elongation	87% DD presented lower crystallinity and mechanical properties, but higher swelling index than 75% or 96% DD	[79]
Nanofibers	59, 76, 85	Thermal degradation	Thermal stability decreased with increase in DD	[80]
Films	15–70	Tensile strength, % elongation	Tensile strength, and % elongation increased with increase in DD	[81]
Sponge	58, 73, 82, 88, 91	Swelling index, tensile strength	Swelling index increased and tensile strength decreased with increase in DD	[82]
Films	72–85	Crystallinity, tensile strength, % elongation, degradation rate	Crystallinity increased and tensile strength decreased with increase in DD, % elongation decreased when the DD was increased from 72% to 75% and decreased when it increased from 75% to 85%. No difference in degradation rates	[83]
Beads	83,94,96	Tensile strength, thermal degradation	Tensile strength, thermal stability increased with increased in DD	[84]

**Table 4:** Surface area, pore diameter and pore volume of CS/mesoporous silica composites and hybrids.

System	Surface Area/m^2^ g^−1^	Pore Size /nm	Pore Volume/cm^3^ g^−1^	Ref
CS	130.2	3.98	0.482	[331]
CS/silica (electrospun)	272.3	3.52	0.431	
SBA-15	809.4	6.6	1.10	[332]
SBA-15 (10% CS)	653.9	6.6	0.90	
SBA-15 (20% CS)	461.9	6.7	0.80	
CS	150	-	0.753	[312]
CS/silica (43% CS)	342	-	1.092	
Silica	739	-	3.645	
SBA-15	876	7.8	1.30	[333]
SBA-15/CS/Fe_2_O_3_	446	6.7	0.90	
CS/silica (81.3% Si)	357.3	8.18	0.730	[325]
CS/silica (74.4% Si)	309.7	6.19	0.479	
CS/silica (59.9% Si)	268.1	6.08	0.407	

**Table 5:** Adsorption performance of CS/GO composites in the removal of heavy metal ions.

Adsorbent	Adsorbate	pH	Ads. Cap./mg g−1	Kinetic Model	Isotherm Model	Ref
CS/GO-SH	Cd(II)	5.0	177	PSO	Freundlich	[339]
	Pb(II)	5.0	447	PSO	Freundlich	
	Cu(II)	5.0	425	PSO	Freundlich	
CS/GO	Cu(II)	6.0	254	PSO	Langmuir	[195]
CS/GO aerogel	Cu(II)	6.0	407	PSO	Langmuir	
CS/GOnano-fibrous	Pb(II)	6.0	461	Double-exp	R–P	[337]
	Cu(II)	6.0	423	Double-exp	R–P	
	Cr(VI)	3.0	310	Double-exp	R–P	
MCS/GO with EDTA	Pb(II)	5.0	206	PSO	Langmuir	[336]
	Cu(II)	5.5	207	PSO	Langmuir	
	As (III)	8.0	43	PSO	Freundlich	
MCS/GO	Cu(II)	7.0	217	PSO	Langmuir	[340]
MCS/GO	As(III)	7.3	2.3	PSO	Langmuir	[341]
CS/GO/MOF	Cr(VI)	3.0	144	PSO	Langmuir	[217]
MCS/GO	Cr(VI)	2.0	270	PSO	Langmuir	[342]
MSC/GO with IL	Pb(II)	5.0	85	PSO	Langmuir	[343]
MSC/GO gel beads	Cd(II)	6.0	86	PSO	Langmuir	[228]
	Pb(II)	5.0	189	PSO	Langmuir	
	Cu(II)	5.0	55	PSO	Langmuir	
MSC/3D-GO	Pb(II)	8.5	957	-	-	[344]
CS-GO/CMC aerogel	Cr(VI)	-	127	-	Langmuir	[230]
MCS/GO	Cr(VI)	2.0	100	PSO	Freundlich	[345]
CS/GO	U(VI)	6.0	78	PSO	Freundlich	[346]
MCS/3D graphene	Pb(II)	8.5	947	-	-	[344]
CS/GO-PVA	Cd(II)	8.0	172	PSO	Langmuir	
CS/GO-PVA	Ni(II)	8.0	71	PSO	Langmuir	[347]
MCS/GO-EDTA	Pb(II)	8.3	666	PFO	Langmuir	[172]
CS/GO gel	Pb(II)	6.0	470	PSO	Langmuir	[348]
CS/GO-silica	Pb(II)	6.0	256	PSO	Langmuir	[225]

**Table 6:** Adsorption performance of CS/CNT, CS/BC and CS/AC composites in the removal heavy metal ions.

Adsorbent	Adsorbate	pH	Ads. Cap./ mg g^−1^	Kinetic Model	Isotherm Model	Ref
CS/CNT/ CoFe_2_O_4_	Pb(II)	6.0	140	PSO	Langmuir	[245]
MCS/CNT	Pb(II)	5.0	101	PSO	Sips	[246]
CS/CNT/PDA	Cu(II)	7.0	112	PSO	Langmuir	[233]
CS/CNT	Cu(II)	7.0	115	PSO	Langmuir	[349]
CS/CNT at 293 K	Cr(VI)	2.0	142	PSO	Langmuir	[350]
at 303 K	Cr(VI)	2.0	151	PSO	Langmuir	
at 313 K	Cr(VI)	2.0	164	PSO	Langmuir	
CS/CNT/PB	Cs(I)	6.0	219	PSO	Freundlich	[249]
	Sr(II)	6.0	205	PSO	Freundlich	
CS/CNT	U(VI)	4.0	126	PSO	Langmuir	[351]
MCS/CNT	Cr(III)	4.0	66	PSO	Langmuir	[352]
	Cr(VI)	4.0	449	PSO	Langmuir	
CS/BC/ PMDA	Cu(II)	5.0	96	PSO	Langmuir	[261]
	Pb(II)	5.0	13	PSO	Langmuir	
	Cd(II)	5.0	38	PSO	Langmuir	
CS/BC/β-CD	Cr(VI)	2.0	206	PSO	Freundlich	[263]
MCS/BC	Cr(VI)	3.0	30	PSO	Freundlich	[353]
	Cu(II)	5.8	54	PSO	Freundlich	
CS/BC	Hg(II)	3.0	594	PSO	Langmuir	[258]
	Pb(II)	5.0	210	PSO	Langmuir	
CS/BC/PAA	Mn(II)	3–7	139	PSO	Langmuir	[262]
	Co(II)	3–7	135	PSO	Langmuir	
	Pb(II)	3–7	476	PSO	Langmuir	
CS/BC/Clay	Cu(II)	5.0	121	Elovich	Freundlich	[354]
	Pb(II)	5.0	336	PSO	Temkin	
	Zn(II)	5.0	134	Elovich	Freundlich	
CS/AC	Zn(II)	6.0	60	-	Langmuir	[281]
CS/AC	Cr(VI)	5.0	84	PSO	Langmuir	[282]
CS/AC	Pb(II)	5.0	125	PFO	Freundlich	[287]
	Cd(II)	5.0	69	PFO	Freundlich	
CS/PEO/AC	Fe(III)	3.0	217	-	Langmuir/	[355]
	Cu(II)	5.0	195	-	Freundlich	
CS/AC	Hg(II)	7.0	576	-	Langmuir	[289]
CS/AC/PVA	Cr(VI)	2.0	109	PSO	Langmuir	[293]

Abbreviations: PMDA: pyromellitic dianhydride; PAA: poly(acrylic acid); PVA: poly(vinyl alcohol); PEO: poly(ethylene oxide); PB: Prussian blue.
